# Multilayered PdSe_2_/Perovskite Schottky Junction for Fast, Self‐Powered, Polarization‐Sensitive, Broadband Photodetectors, and Image Sensor Application

**DOI:** 10.1002/advs.201901134

**Published:** 2019-08-07

**Authors:** Long‐Hui Zeng, Qing‐Ming Chen, Zhi‐Xiang Zhang, Di Wu, Huiyu Yuan, Yan‐Yong Li, Wayesh Qarony, Shu Ping Lau, Lin‐Bao Luo, Yuen Hong Tsang

**Affiliations:** ^1^ Department of Applied Physics The Hong Kong Polytechnic University Hung Hom Kowloon Hong Kong 999077 China; ^2^ School of Electronic Science and Applied Physics Hefei University of Technology Hefei Anhui 230009 China; ^3^ School of Physics and Engineering and Key Laboratory of Material Physics of Ministry of Education Zhengzhou University Zhengzhou Henan 450052 China

**Keywords:** image sensors, palladium diselenide, perovskites, photodetectors, polarization‐sensitive

## Abstract

Group‐10 transition metal dichalcogenides (TMDs) with distinct optical and tunable electrical properties have exhibited great potential for various optoelectronic applications. Herein, a self‐powered photodetector is developed with broadband response ranging from deep ultraviolet to near‐infrared by combining FA_1−_ *_x_*Cs*_x_*PbI_3_ perovskite with PdSe_2_ layer, a newly discovered TMDs material. Optoelectronic characterization reveals that the as‐assembled PdSe_2_/perovskite Schottky junction is sensitive to light illumination ranging from 200 to 1550 nm, with the highest sensitivity centered at ≈800 nm. The device also shows a large on/off ratio of ≈10^4^, a high responsivity (*R*) of 313 mA W^−1^, a decent specific detectivity (D*) of ≈10^13^ Jones, and a rapid response speed of 3.5/4 µs. These figures of merit are comparable with or much better than most of the previously reported perovskite detectors. In addition, the PdSe_2_/perovskite device exhibits obvious sensitivity to polarized light, with a polarization sensitivity of 6.04. Finally, the PdSe_2_/perovskite detector can readily record five “P,” “O,” “L,” “Y,” and “U” images sequentially produced by 808 nm. These results suggest that the present PdSe_2_/perovskite Schottky junction photodetectors may be useful for assembly of optoelectronic system applications in near future.

## 1 Introduction

Organic/inorganic halide perovskite materials have in the past decade stimulated broad research interest for their excellent optoelectronic properties including long diffusion length, low trapping density, large absorption coefficient, and multiband light absorption.^1^, ^2^, ^3^, ^4^, ^5^ With these distinct properties, this group of hybrid halide materials has demonstrated great potential as building blocks for assembly of various optoelectronic devices, including light‐emitting diodes,^6^, ^7^ lasers,^8^, ^9^ solar cells,^10^ and photodetectors.^11^, ^12^ Among these optoelectronic devices, photodetectors are of great importance for their promising applications in both fundamental science and industrial fields, such as image sensing, optical communication, night‐vision, and environmental monitoring.^13^, ^14^, ^15^ To date, a number of emerging perovskite materials have been extensively explored for various photodetector device applications.^4^, ^16^, ^17^, ^18^ For example, Song and co‐workers reported the large‐scale fabrication of CH_3_NH_3_PbI_3_ nanowires (NWs) by a one‐step self‐assembly approach. The as‐assembled CH_3_NH_3_PbI_3_ NWs photodetector exhibited a responsivity of 1.32 A W^−1^ and a specific detectivity of 2.5 × 10^12^ Jones, with a response time of 0.3 ms.^19^ Additionally, Deng et al. developed highly sensitive perovskite photodetectors made of high‐quality single‐crystalline aligned CH_3_NH_3_PbI_3_ microwire arrays through facile blade coating approach. The resulting micro‐photodetectors showed broadband sensitivity with peak responsivity (13.57 A W^−1^) and detectivity (5.25 × 10^12^ Jones) under 420 nm light illumination. What is more, the rise and fall time were calculated to be 80 and 240 µs, respectively.^20^ Despite these great achievements, it is undeniable that most of the perovskite photodetectors are often characterized by low specific detectivity and slow photoresponse, which inevitably limit their practical applications, especially in high‐capacity networks, high‐speed telecommunication, and fast imaging.^4^, ^21^ To address the above issues, a number of new device geometries that are fabricated by combining perovskite and other materials (e.g., PbSe quantum dots^22^ graphene,^4^ and black phosphorus^23^) have been proposed, which proves to be an excellent strategy to improve the perovskite based detectors performance.^24^

As a new material family, 2D layered transition metal dichalcogenides (TMDs) materials have shown great potential for electronics and optoelectronics applications due to their unique thickness‐dependent properties, high carrier mobility, and good air stability.^25^, ^26^, ^27^ Owing to these outstanding properties, 2D layered TMDs (e.g., MoS_2_,^16^ WS_2_,^28^ and PtSe_2_ 29) have been successfully integrated with perovskite to achieve high‐performance photodetectors including photoconductor, phototransistor, and photodiode. By virtue of synergistic effect, these detectors exhibited enhanced photoconductive gain and photoresponsivity. Enlightened by the above works, we developed a high‐performance Schottky junction photodetector by combining Cs‐doped FAPbI_3_ perovskite with multilayer PdSe_2_, a newly explored 2D layered group‐10 TMDs, which has been widely applied in solar cells, field‐effect transistors, and photodiodes.^30^, ^31^, ^32^ Such a PdSe_2_/Cs‐doped FAPbI_3_ perovskite offers obvious advantages in the following three aspects: i) Compared with other 2D materials like black phosphorus, the wafer‐scale and uniform PdSe_2_ films with precise layer numbers can be synthesized on arbitrary substrates in low temperature, which renders the easy fabrication of large‐area integrated devices. ii) It has been theoretically reported that 2D PdSe_2_ exhibits high carrier mobility more than 1000 cm^2^ V^−1^ s^−1^,^33^, ^34^, ^35^ resulting in fast photoresponse.^29^ iii) Significantly, its low‐symmetry crystal structure endows PdSe_2_ based device with good capability to detect polarized light signal.^33^ It is revealed that the as‐assembled PdSe_2_/perovskite device exhibits obvious photovoltaic behavior, which enables it to function as a self‐powered photodetector for detecting light over broadband wavelength region from 200 to 1550 nm. Under 808 nm light illumination, the device achieves impressive device performance in terms of a large on/off ratio of ≈10^4^, a high responsivity (*R*) of 313 mA W^−1^, a decent specific detectivity (D*) of ≈10^13^ Jones, a high polarization sensitivity of 6.04, as well as a fast response speed of 3.5/4 µs. These results suggest that the present device may find potential application in future optoelectronic devices and systems.

## 2 Results and Discussion

**Figure 1:**
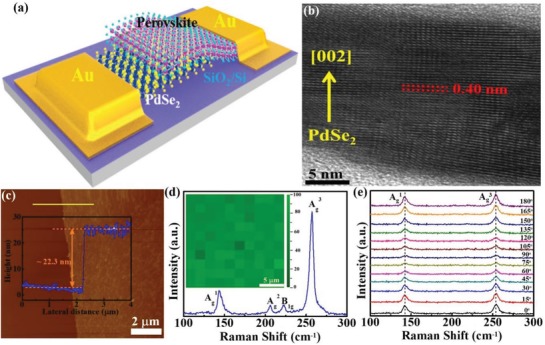
a) Schematic illustration of the as‐fabricated PdSe_2_/perovskite device. b) Typical cross‐section TEM image of PdSe_2_. c) AFM image and inset shows height profile. d) The Raman spectrum of PdSe_2_ sample. Inset shows 2D Raman intensity mapping of A_g_^3^ mode within 20 **×** 20 µm^2^. e) Angle‐resolved polarized Raman spectra of 2D PdSe_2_.

**Figure** **1**a shows the schematic illustration of the PdSe_2_ layer/perovskite Schottky junction device. In this study, the large‐area, continuous, and high‐quality multilayer 2D PdSe_2_ films were synthesized on a SiO_2_/Si substrate (300 nm SiO_2_ thickness) via a simple selenization method,^36^ and the Cs‐doped FAPbI_3_ perovskite layer as the light absorption medium was then drop casted by spin‐coating method (More details about the synthesis of PdSe_2_, perovskite, and the device fabrication are provided in the Experimental Section). Due to distinct difference in contrast, both PdSe_2_ layer and perovskite material can be easily distinguished from photographic device image, as shown in Figure S1a in the Supporting Information. From the cross‐section transmission electron microscopy (TEM) image in Figure 1b, we can easily find that the PdSe_2_ films are multilayered structure with a thickness of about 22 nm, in consistence with the result derived from atomic force microscopy (AFM) (Figure 1c). The PdSe_2_ sample crystallizes in pentagonal manner with lattice distance of 0.27 and 0.23 nm, corresponding to the (200) and (022) planes, as shown in Figure S1b in the Supporting Information.^30^, ^34^ The chemical composition was confirmed by an energy dispersive spectroscopy analysis and X‐ray photoemission spectroscopy (Figure S1c–e, Supporting Information), in which the stoichiometric ratio of Se: Pd is determined to be 2: 1. Figure 1d presents Raman spectrum of 2D PdSe_2_, which consists of four obvious peaks at ≈143.2, ≈206.1, ≈223.2, and ≈257.2 cm^−1^. The first three peaks defined as A_g_^1^, A_g_^2^, and B_1g_ could be ascribed to the movements of Se atoms, while the highest mode (A_g_^3^) at the highest wavenumber is assigned to the relative movements between Se and Pd atoms.^34^, ^37^ Further Raman intensity mapping of A_g_^3^ mode in inset of Figure 1d shows a narrow peak intensity distribution, signifying the good uniformity and homogeneity of as‐grown PdSe_2_ films. To study the anisotropic property of 2D PdSe_2_, the angle‐resolved polarized Raman spectroscopy was measured by adjusting the analyzer to be parallel to laser polarization. As presented in Figure 1e, compared with stronger Raman peaks of A_g_^1^ and A_g_^3^, the A_g_^2^ and B_1g_ peaks are too weak to be observed in the all polarized Raman spectra due to the lower excitation light intensity. Specifically, the peak intensity of A_g_^1^ and A_g_^3^ mode is found to gradually decrease in the polarization angle ranging from 0° to 90° but increase when the polarization angle varies from 90° to 180°, as revealed by the anisotropy variation period of two Raman modes (A_g_^1^ and A_g_^3^) in Figure S2 in the Supporting Information. According to the field emission scanning electron microscopy (FESEM) image in Figure S3a in the Supporting Information, the large‐area and continuous Cs‐doped FAPbI_3_ perovskite films with relatively smooth surface can be easily synthesized. From the X‐ray diffraction (XRD) patterns in Figure S3b in the Supporting Information, all the signals can be readily ascribed to the diffraction peaks of black phase FAPbI_3_ perovskite.^18^, ^38^

**Figure 2:**
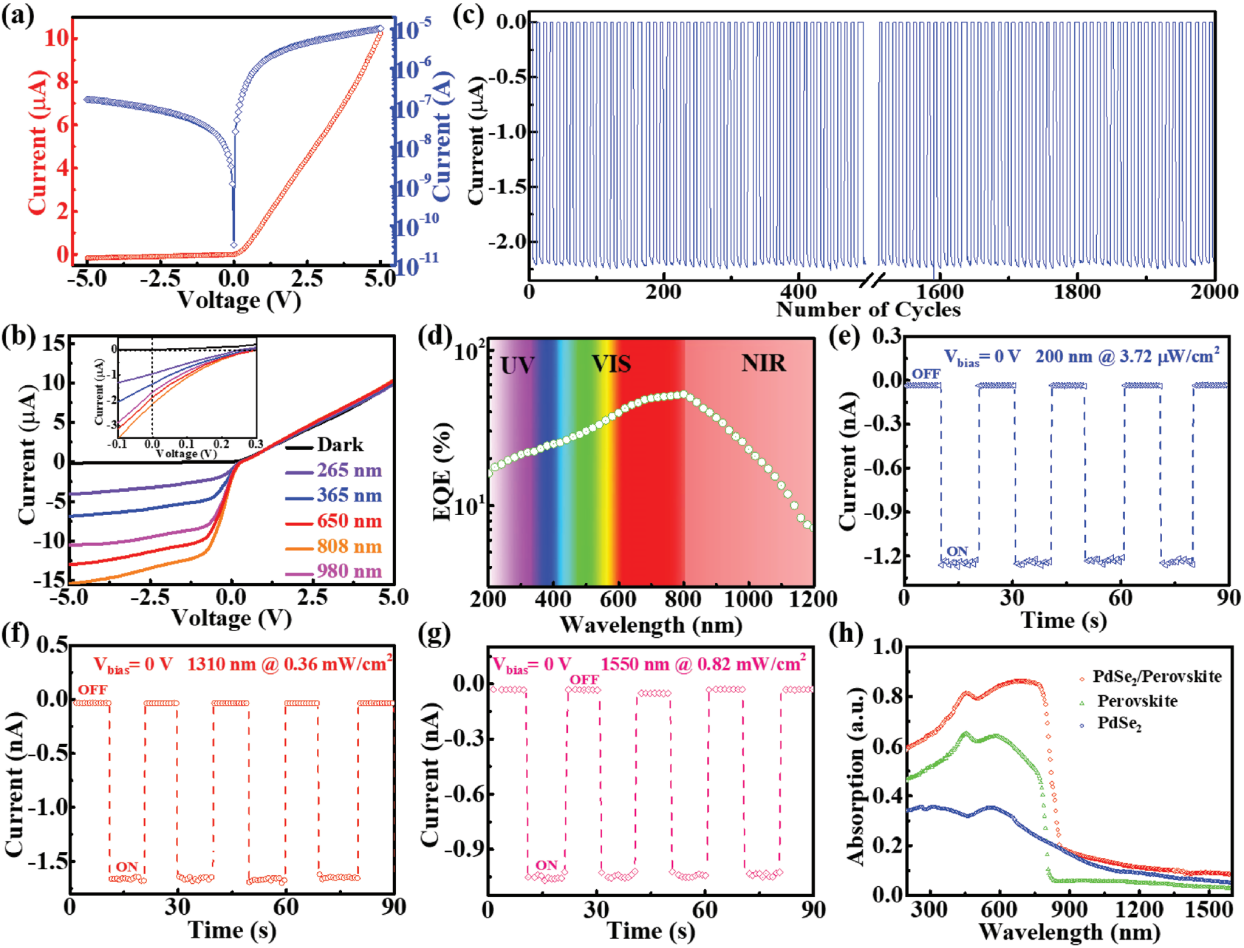
a) *I–V* curve of the PdSe_2_/perovskite Schottky junction device in dark. b) *I–V* curve of photodetector in dark and under different light illuminations with wavelength of 265, 365, 650, 808, and 980 nm (Intensity: 0.3 mW cm^−2^), respectively. c) Time‐dependent response of Schottky junction device after 2000 cycles operation. d) The wavelength‐dependent external quantum efficiency (EQE) of PdSe_2_/perovskite device at zero bias under the same light intensity of 10 µW cm^−2^. Temporal photoresponse of Schottky junction device under e) 200 nm (3.72 µW cm^−2^), f) 1310 nm (0.36 mW cm^−2^), and g) 1550 nm (0.82 mW cm^−2^) light illuminations. h) Absorption spectrum of the PdSe_2_/perovskite hybrid system. The absorption spectra of pure PdSe_2_ and perovskite prepared on quartzes are also measured for comparison.

**Figure** **2**a plots the current–voltage (*I–V*) curve of device in dark. Apparently, this Schottky junction displays a conventional rectifying behavior with a rectification ratio of ≈60 at ± 5 V, which is higher than that of MoS_2_/WS_2_ (≈5),^39^ PtSe_2_/MoS_2_ (15–20),^40^ graphene/In_2_S_3_ (≈32),^41^ and PtSe_2_/perovskite heterojunctions (≈10),^29^ but is slightly lower than that of MoS_2_ p–n junction (≈67).^42^ Considering the good linear *I–V* characteristics for Au‐PdSe_2_ and Au‐perovskite achieved (Figure S4, Supporting Information), the observed rectifying behavior should arise from the Schottky barrier formed at the PdSe_2_/perovskite interface. Interestingly, when the device is shinned by several light sources (265, 365, 650, 808, and 980 nm) with constant light intensity of 0.3 mW cm^−2^, the photocurrent at negative bias region substantially increases with increasing wavelength from 265 to 808 nm but decreases subsequently when the incident wavelength further increases to 980 nm (Figure 2b), indicative of a peak sensitivity in the region from 650 to 980 nm. Careful examination of *I–V* curves finds a weak photovoltaic effect under light illumination (the inset of Figure 2b). Despite relatively low energy‐conversion efficiency with a short‐circuit current (*I*_sc_) of ≈2.14 µA and an open‐circuit voltage (*V*_oc_) of ≈0.3 V, the weak photovoltaic behavior can enable the present device to function as a self‐powered photodiode operating without external bias voltage, producing obvious photoresponse in Figure 2c. Further photoresponse analysis reveals that the PdSe_2_/perovskite photodiode can be easily switched between on and off states with perfect reproducibility and stability by repeatedly turning light (808 nm) on and off, even after 2000 cycles of operation, yielding a high on/off ratio of 6.46 × 10^4^. From the wavelength‐dependent external quantum efficiency (EQE) plotted in Figure 2d, one can easily find that the device displays a good detection capability covering from 200 to 1200 nm, with the peak EQE located at ≈800 nm (Figure 2d), in consistence with the absorption curve shown in Figure 2h. Although the sensitivity of PdSe_2_/perovskite Schottky junction was relatively weaker in the deep ultraviolet and NIR region, the device is still capable of detecting 200 nm (deep UV), 1310 nm (O band), and 1550 nm (C band) light illumination (Figure 2e–g), which are potentially important for application in both military surveillance and optical communication.^43^, ^44^, ^45^ We believe such a broadband photoresponse with good spectral selectivity is directly associated with optical absorption of hybrid PdSe_2_/perovskite system. Compared with the individual absorption spectrum of perovskite on a quartz substrate (Figure 2h), the absorption spectrum of hybrid PdSe_2_/FA_0.85_Cs_0.15_PbI_3_ system also covers the NIR region larger than 800 nm (the inherent cutoff optical absorption of perovskite), due to the strong absorption of PdSe_2_ films in NIR region.

**Figure 3:**
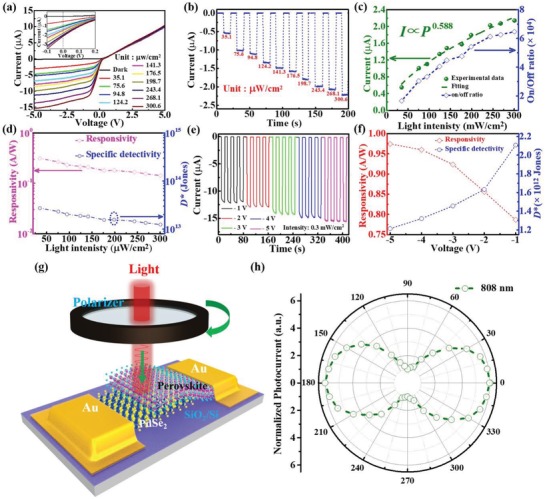
a) *I–V* curves of the PdSe_2_/perovskite detector in dark and under 808 nm light irradiation with different intensities. b) The real time‐dependent photocurrent of device under 808 nm light illumination with different light intensities. c) Photocurrent and on/off ratio as a function of light intensity at 0 V. d) *R* and D* as a function of incident power intensity. e) The real time‐dependent photocurrent of device at reverse bias under 808 nm light illumination (0.3 mW cm^−2^). f) *R* and D* as a function of operating bias voltage under light intensity of 0.3 mW cm^−2^. g) Schematic diagram of polarized detection device based on the PdSe_2_/perovskite. h) The evolution photocurrent as a function of different polarized angle.

The photoresponse properties of Schottky junction photodiode are found to highly depend on the NIR light (808 nm) intensity. **Figure** **3**a depicts a series of *I–V* curves under 808 nm light illumination with different intensities. It is obvious that the photocurrent will rise sharply at both zero and reverse bias with the increase of power intensity. Specifically, the photocurrent increases from 0.549 to 2.14 µA at 0 V with light intensity changing from 35.1 to 300.6 µW cm^−2^ (Figure 3b). Such an evolution in photoresponse is understandable given the fact that the increased population of photoexcited electron–hole pairs can lead to a monotonous increase in photocurrent under higher light intensity. The above light intensity dependent photocurrent can be described using formula of *I*_ph_ ∝ *P^θ^*, and then θ is determined to be 0.588 (*θ* < 1), which indicates the presence of traps and defects in photodetector.^44^, ^46^, ^47^ As a matter of fact, a similar evolution was observed in the dependence of on/off ratio on light intensity as well: The on/off ratio is found to increase from 1.66 × 10^4^ to 6.46 × 10^4^ when the light intensity ranges from 35.1 to 300.6 µW cm^−2^.

To quantitatively evaluate the performance of Schottky junction photodetector, two representative parameters including responsivity (*R*) and specific detectivity (D*) were determined using extensively studied equations as follows

(1) $$R(AW^{-1})=\frac{I_{\mathrm{ph}}}{P_{\mathrm{opt}}S}=\frac{I_{\mathrm{light}}-I_{\mathrm{dark}}}{P_{\mathrm{opt}}S}$$

(2) $$D*=A^{1/2}R/{\left(2qI_{d}\right)}^{1/2}$$

where the net photocurrent (*I*_ph_) can be determined by the difference between photocurrent (*I*_light_) and dark current (*I*_dark_), *P*_opt_ is the incident power intensity, *S* is the effective illuminated area (0.05 cm^2^), *A* is the effective device area (0.08 cm^2^), and *q* is the elementary electronic charge. Based on the above two equations, the *R* and D* were calculated to be 313 mA W^−1^ and 2.72 × 10^13^ Jones by adopting lower light intensity of 35.1 µW cm^−2^ at 0 V. These values at different light intensities were plotted in Figure 3d, from which both *R* and D* are found to decrease with increasing power intensity. Such decrease at high light intensity is ascribed to enhanced carrier recombination by defects.^43^, ^45^ Actually, apart from the influence of light intensity, the photoresponse is also dependent on the operating bias voltage. As depicted in Figure 3e, as the operating voltage rises from −1 to −5 V, the photocurrent monotonously increases from 11.8 to 14.8 µA. Meanwhile, both corresponding *R* and D* values under different operating voltages were calculated and plotted in Figure 3f. One can clearly find that *R* increases gradually with increasing bias voltage. Such a finding is reasonable as the separation efficiency and drift velocity of the photogenerated carriers are improved by enhanced built‐in electric field at reverse bias. On the contrary, the D* is found to slightly decrease with the increase of working bias voltage owing to the significantly enhanced dark current at reverse bias. In addition to the superior photoresponse characteristics, the PdSe_2_/perovskite detector also exhibits high sensitivity to polarized incident light, which is often observed in some low‐dimensional nanostructure‐based photodetectors.^48^, ^49^ Figure 3g shows the testing setup of photoresponse as a function of incident light with different polarization angle, which was obtained by a homemade polarization detection test equipment (The light can pass through a polarizer to produce polarized light illuminating device). Figure 3h plots the angle‐resolved normalized photocurrent curve under 808 nm light illumination at 0 V, from which one can clearly see that the output photocurrent highly depends on polarization angle. When the polarization angle changes from 0° to 360°, the normalized photocurrent varies periodically and reaches the maximum value at 0° (180°) and minimum value at 90° (270°), with a high polarization sensitivity of 6.04. Such a high value can be directly ascribed to high‐quality and strong anisotropic crystal structure of 2D PdSe_2_,^30^, ^33^ which holds potential application in optical switch, navigation, and high contrast polarizer.^50^

**Figure 4:**
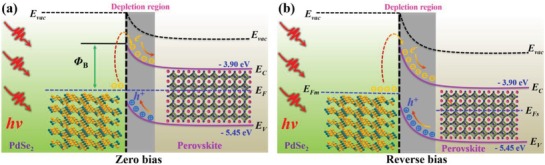
Energy band diagram of PdSe_2_/perovskite Schottky junction under a) zero and b) reverse bias.

**Figure 5:**
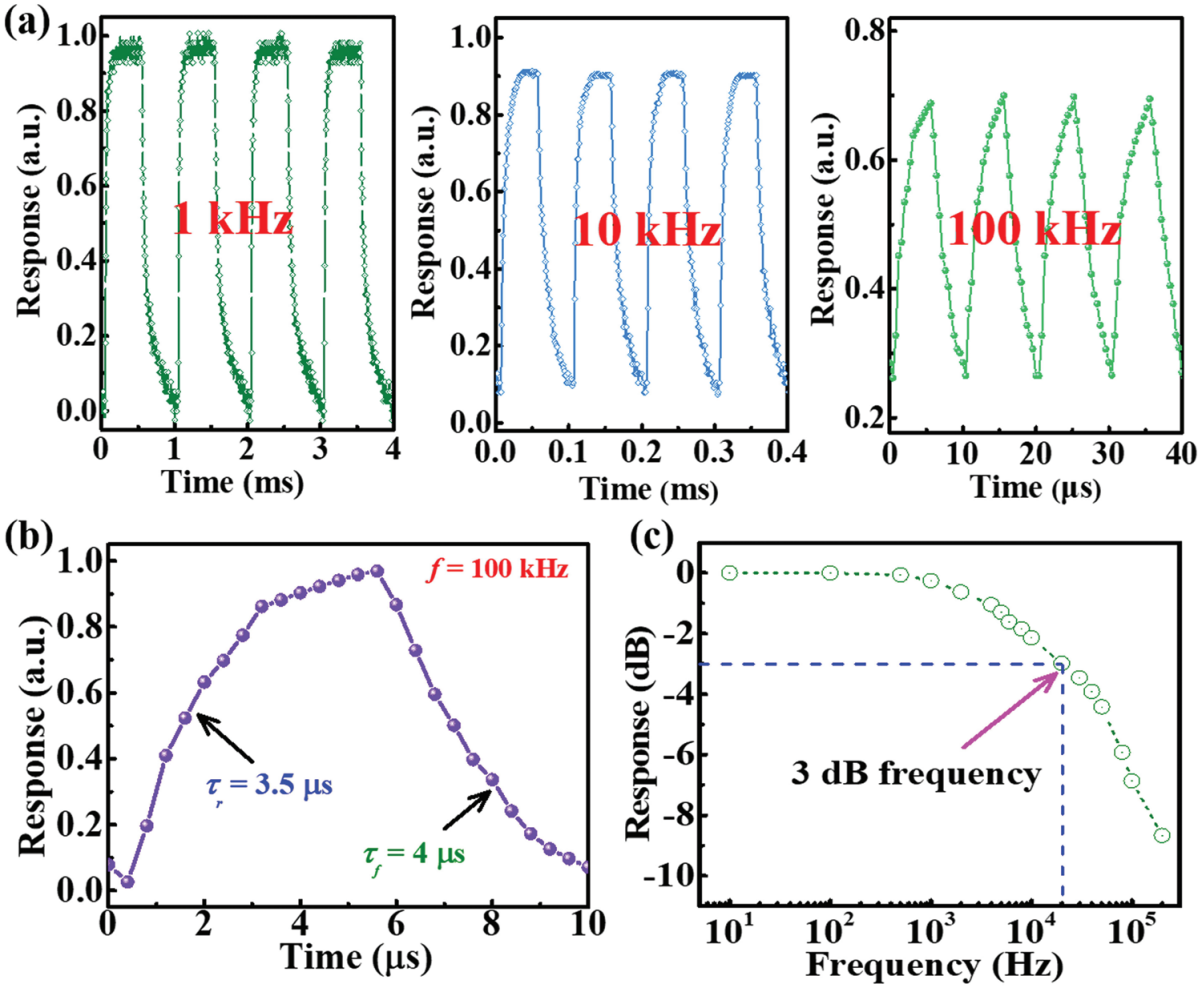
a) Photoresponse of the PdSe_2_/perovskite Schottky junction device under pulsed light illumination with frequency of 1, 10, and 100 kHz. b) A single enlarged curve of photoresponse for calculating rise/fall time. c) Normalized response versus varying frequency, showing 3 dB cutoff frequency of ≈20.1 kHz.

The obvious photoresponse mentioned above can be explained by the energy band diagram shown in **Figure** **4**a,b. Based on the ultraviolet photoemission spectroscopy (UPS) analysis in Figure S5a in the Supporting Information, the multilayer PdSe_2_ is semimetal with work function of ≈5.12 eV. In addition, according to the photoluminescence (PL) spectrum (Figure S5b, Supporting Information), the bandgap of the perovskite is estimated to be ≈1.55 eV. By combining with UPS results in Figure S5c in the Supporting Information, the Fermi level, bottom level of conduction band, and onset of valence band of FA_0.85_Cs_0.15_I_3_ are determined to be at ≈4.57 eV, ≈3.9 eV, ≈5.45 eV, indicating a weak *n*‐type semiconducting characteristic.^18^, ^21^ Once the perovskite and PdSe_2_ are in contact, owing to the difference in Fermi level, the electrons would move from the perovskite to the PdSe_2_ until both Fermi levels align, leading to the bending upward of energy levels and the formation of built‐in potential at the interface of PdSe_2_/perovskite. As illustrated in **Figure** **5**a, when the photon energy for incident light is larger than the bandgap of perovskite (*E*_g_ = 1.55 eV), the light will be absorbed by both perovskite and PdSe_2_ films, the resultant electron–hole pairs will be excited and then quickly separated by the built‐in potential, give rising to photocurrent.^29^ For the photons with energy between bandgap of perovskite (wavelength >800) and Schottky junction barrier (*Φ*_B_ *< hν < E*_g_), the PdSe_2_ films will mainly absorb the light. Electrons generated from PdSe_2_ will overcome contact barrier and move to the perovskite, which endows the PdSe_2_/perovskite device with ability to detect the NIR light with wavelength up to 1550 nm. As a matter of fact, the depletion region could be further broadened by reverse bias, allowing the larger population of photoexcited carriers to generate higher photocurrent. The lower PL intensity of the hybrid PdSe_2_/perovskite system also suggests the efficient separation of photoexcited carriers, compared with PL performance from individual perovskite (Figure S5b, Supporting Information).

Next, we studied the response time of PdSe_2_/perovskite device, which is an important criterion for optical communication application.^51^ To record the response speed, an NIR laser diode driven by a function generator was used as the pulsed illuminating source. Figure 5a displays the response of detector to pulsed signal with frequency of 1, 10, and 100 kHz, signifying that the as‐assembled Schottky junction device can work properly with excellent stability and reproducibility at very high frequency. Based on the definition of response speed, the rise/fall time (τ_r_/τ_f_) is determined to be 3.5/4 µs from the single magnified response curve at 100 kHz (Figure 5b).^52^, ^53^, ^54^, ^55^ Such a fast response speed is correlated to the built‐in potential at the interface of PdSe_2_/perovskite and the outstanding electrical properties of PdSe_2_, which will be discussed in detail later. Figure 5c shows the normalized response versus input pulsed frequency, from which the 3 dB bandwidth of the Schottky junction device is estimated to be ≈20.1 kHz. Such 3 dB frequency is much higher than that of SnS_2_/PbS hybrid detector (≈50 Hz),^56^ and PtSe_2_/CdTe (≈6.2 kHz),^57^ but slightly lower than that of Sn‐containing perovskite NIR photodetector (≈100 kHz).^58^ **Table** **1** summarizes the main device parameters of the PdSe_2_/perovskite detector and other devices with similar configurations. It is obvious that the response speed, on/off ratio, and specific detectivity are better than not only PdSe_2_ based devices, but also other photodetectors composed of 2D materials.^59^, ^60^ Such good results can be mainly attributed to the following three factors: 1) The high quality PdSe_2_/perovskite interface. The relatively low amounts of defects can greatly reduce the recombination activity, which will greatly increase responsivity as well as specific detectivity. (2)) The strong built‐in potential formed by Schottky junction. The built‐in electric field is highly beneficial for the fast separation and transportation of photogenerated electron–hole carriers, giving rise to fast photoresponse. 3) The relatively good carrier transport property 2D PdSe_2_. According to our calculation (Figure S6, Supporting Information), the hole mobility is calculated to be 4.75 cm^2^ V^−1^ s^−1^, which enables quick drift of charge carriers under the built‐in potential, leading to short carrier transit times and then quick response speed.

**Table 1:** Comparison of the performance parameters of the PdSe_2_/perovskite detector with similar heterostructure photodetectors

Device structure	τ_r_/τ_f_	*I*_light_ */I*_dark_	D* (Jones)	Self‐powered	λ [µm]	Ref.
PdSe_2_/FA_0.85_Cs_0.15_PbI_3_	3.5/4 µs	≈10^4^	≈10^13^	Yes	0.2–1.5	This work
MoS_2_/MAPbI_3_	10.7/6.2 s	10	≈10^10^	No (20 V)	0.52–0.85	16
WS_2_/CH_3_NH_3_PbI_3_	2.7/7.5 ms	≈10^4^	≈10^12^	No (5 V)	0.47–0.627	24
PdSe_2_/MoS_2_	74.5/93.1 ms	<10	≈10^9^	No (1 V)	0.45–10.6	32
PdSe_2_ FET	220/220 ms	<10	≈10^9^	No (1 V)	–	33
WSe_2_/CH_3_NH_3_PbI_3_	–	–	≈10^11^	No (2 V)	0.2–0.9	59
MoS_2_/CsPbBr_3_	0.72/1.01 ms	–	≈10^10^	No (10 V)	0.35–0.65	60

**Figure 6:**
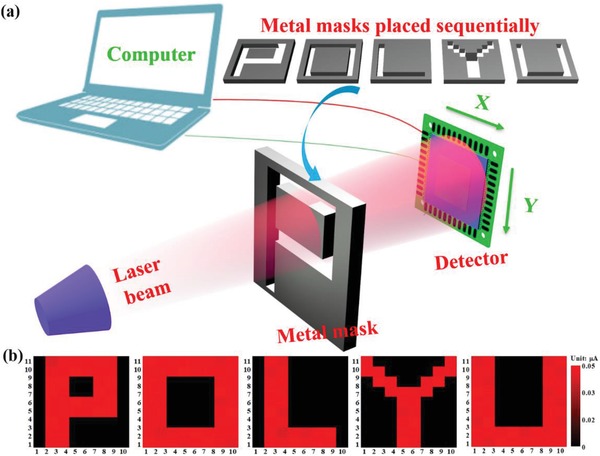
a) Schematic diagram of the experimental setup for detector to realize 808 nm near infrared imaging sensing. b) The corresponding 2D current mapping of characters “P,” “O,” “L,” “Y,” and “U” produced by 808 nm illumination.

As an important optoelectronic device, infrared image sensor that has been widely applied in digital systems, thermometer, surveillance camera, and temperature measurement, has stimulated increasing research interest.^61^, ^62^ To further explore the possibility of the present PdSe_2_/perovskite device for infrared image sensing, an image sensing analysis setup based on individual PdSe_2_/perovskite device was developed, as displayed in **Figure** **6**a. For the sake of convenience, five homemade metal masks (“P,” “O,” “L,” “Y,” and “U”) were placed between laser illumination and device sequentially (See the characters of “POLYU” as an abbreviation for our university of “The Hong Kong Polytechnic University”). The measured dark current and photocurrent, corresponding to the background noise level and light intensity level at each pixel, respectively, were incorporated into a plot and then generated an obvious 2D contrast mapping profile. As illustrated in Figure 6b, for all five characters, only the pixels projected by 808 nm irradiation exhibited sizeable photocurrent of around 0.05 µA, while the rest of the area displayed very weak dark current, suggesting that all the shape of characters “P,” “O,” “L,” “Y,” and “U” can be clearly observed in current contrast mapping, with decent resolution. This image sensing ability, along with the pronounced response speed and high specific detectivity render the present device highly promising for future optoelectronic devices and systems application.

## 3 Conclusion

In conclusion, we have developed a fast, self‐powered, highly polarization‐sensitive, and broadband detector by integrating 2D PdSe_2_ with Cs‐doped FAPbI_3_, which can detect broadband light illumination from 200 to 1550 nm. Further photoresponse characterization reveals that the present device exhibits superior performance in terms of a high on/off ratio (≈10^4^), a large responsivity (313 mA W^−1^) and specific detectivity (≈10^13^ Jones), and a good polarization sensitivity (≈6.04), together with a fast response speed of 3.5/4 µs. In addition to above excellent features, with the help of optical‐assisted displacement platform, the five simple images (e.g., “P,” “O,” “L,” “Y,” and “U”) produced 808 nm can be easily recorded by our present detector. Therefore, it is expected that such a simple, and reliable PdSe_2_/perovskite Schottky junction photodetector will show great potential for hybrid optoelectronic systems.

## 4 Experimental Section

*Materials Synthesis and Characterization*: The large‐area PdSe_2_ films were synthesized via the simple selenization process, as demonstrated in detail in previous studies.^37^ In brief, ≈7 nm Pd layer was deposited on SiO_2_/Si substrate via magnetron sputtering system. Subsequently, the as‐prepared Pd thin films were transformed to PdSe_2_ in tube furnace with Pd coated SiO_2_/Si substrate heated up to 480 °C at center place and Se powder heated up to 220 °C in upstream side of furnace. The Cs‐doped FAPbI_3_ (FA_0.85_Cs_0.15_PbI_3_) perovskite films were prepared based on modified approach previously reported.^21^ The original solution for synthesizing perovskite was prepared by adding 461 mg PbI_2_ (Aldrich, 99%), 38.9 mg CsI (Aldrich, 99.9%), and 145 mg HC(NH_2_)_2_I (FAI) (Aldrich, 99.5%) into a mixed solvent consist of 800 µL of *N*,*N*‐dimethylformamide (99.8%) and 200 µL of dimethyl sulfoxide (>99.9%) and stirred at 80 °C for 1 h. Then, as‐prepared precursor solution was spin coated onto substrate under 4000 rpm and postannealed at 140 °C for 15 min to achieve Cs‐doped perovskite films.

The morphology of Cs‐doped FAPbI_3_ perovskite was investigated by SEM (JEOL Model JSM‐6490). The XRD patterns of perovskite films were carried out to study its crystallinity in a RigakuSmartLab X‐ray diffractometer. The topography of as‐synthesized PdSe_2_ samples was studied by AFM (Benyuan Nanotech Com, CSPM‐4000). Using a field emission transmission electron microscope instrument (JEOL model JEM‐2100F) with an energy‐dispersive spectrometer, the chemical composition and crystal structure of PdSe_2_ were well investigated. The Raman spectrum and mapping of PdSe_2_ sample were performed by a HORIBA Raman spectrometer equipped with a 488 nm argon ion laser. The PL spectrum of perovskite and PdSe_2_/perovskite hybrid were probed by PL system (Edinburgh Photoluminescence).

*Device Fabrication and Analysis*: The PdSe_2_/perovskite Schottky junction device was fabricated by the one‐step spin‐coating method. In short, the perovskite solution was carefully spin coated onto prepatterned substrate to cover exposed parts of PdSe_2_ sample. The overlapped region is effective area for PdSe_2_/perovskite Schottky junction. Parallel Au electrodes (≈50 nm) for perovskite and PdSe_2_ were prepared via magnetron sputtering using homemade shadow mask. The photoelectronic characteristics carried out using a semiconductor analysis system (Keithley 4200‐SCS) together with several lasers diodes with wavelength of 265, 365, 650, 808, 1310, and 1550 nm. To record response speed, the pulsed light signal was provided by laser diode driven by a signal generator (Tektronix, TDS2022B), and an oscilloscope (Tektronix, TDS2012B) was used to record the output photoresponse. For normalized spectral response study, the monochromatic light was generated by a Xe lamp (CEL‐HXF300) equipped with a monochromator (Zolix Instruments, Omni‐nx I).

## Conflict of Interest

The authors declare no conflict of interest.

## Supporting information

Supplementary
