# Supplementary material for: Multilayered PdSe2/Perovskite Schottky Junction for Fast, Self‐Powered, Polarization‐Sensitive, Broadband Photodetectors, and Image Sensor Application
Source: Adv Sci (Weinh). 2019 Aug 7;6(19):1901134. doi: 10.1002/advs.201901134 (PMC6774060; doi:10.1002/advs.201901134)
Supplement: Supplementary file 1 — Supplementary [file ADVS-6-1901134-s001.pdf]

Copyright WILEY-VCH Verlag GmbH & Co. KGaA, 69469 Weinheim, Germany, 2019.

ADVANCED SCIENCE
Open Access

Supporting Information

for *Adv. Sci.,* DOI: 10.1002/advs.201901134

Multilayered $PdSe_2$/Perovskite Schottky Junction for Fast, Self-Powered, Polarization-Sensitive, Broadband Photodetectors, and Image Sensor Application

*Long-Hui Zeng, Qing-Ming Chen, Zhi-Xiang Zhang, Di Wu, Huiyu Yuan, Yan-Yong Li, Wayesh Qarony, Shu Ping Lau, Lin-Bao Luo, and Yuen Hong Tsang**

Supporting information

Multilayered $PdSe_2$/Perovskite Schottky Junction for Fast, Self-Powered, Polarization-Sensitive, Broadband Photodetectors and Image Sensor Application

Long-Hui Zeng,[a] Qing-Ming Chen,[a] Zhi-Xiang Zhang,[b] Di Wu,[c] Huiyu Yuan,[a] Yan-Yong Li,[a] Wayesh Qarony,[a] Shu Ping Lau,[a] Lin-Bao Luo,[b] Yuen Hong Tsang*[a]

[a] Department of Applied Physics, The Hong Kong Polytechnic University, Hung Hom, Kowloon, Hong Kong, China

[b] School of Electronic Science and Applied Physics, Hefei University of Technology, Hefei, Anhui 230009, China

[c] School of Physics and Engineering and Key Laboratory of Material Physics of Ministry of Education, Zhengzhou University Zhengzhou, Henan 450052, China

Corresponding author: yuen.tsang@polyu.edu.hk

Author Contributions

The first two authors contribute this work equally.

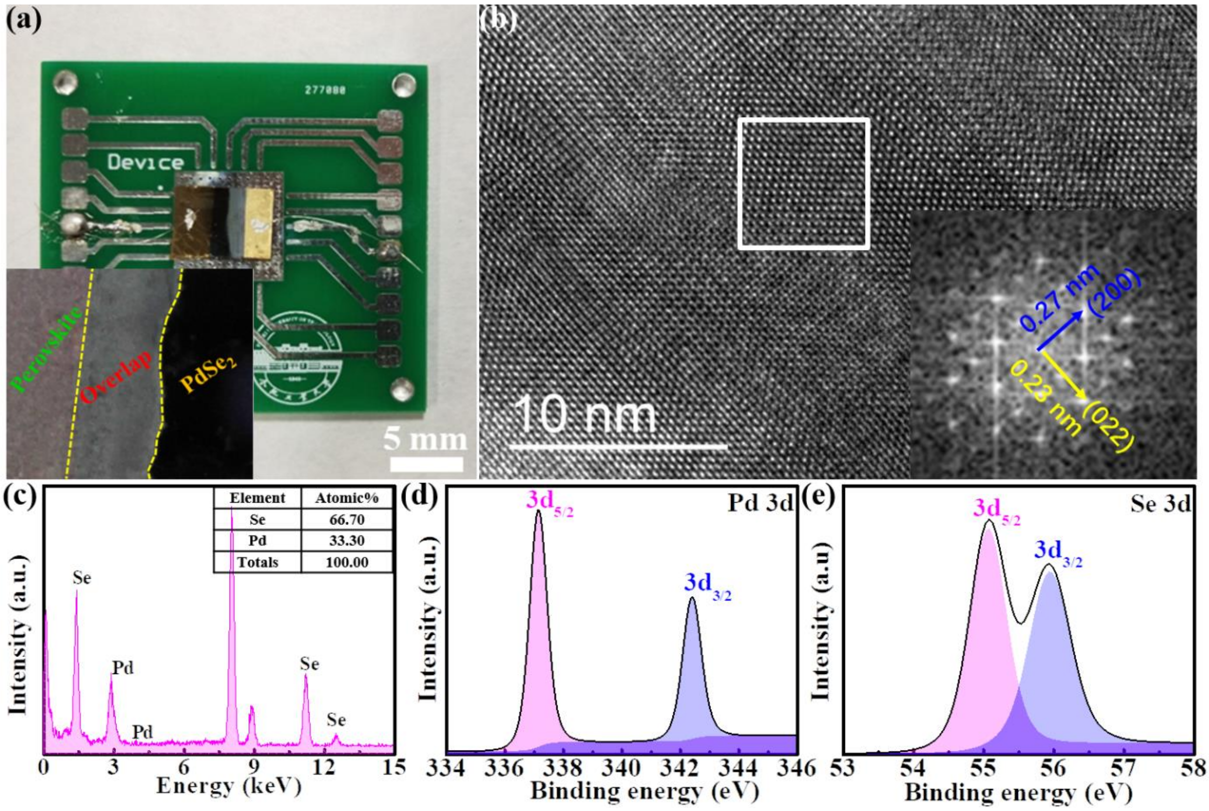

Figure S1 (a) Camera photography of the as-fabricated $PdSe_2$/perovskite device. (b) The HRTEM image of $PdSe_2$ films and the inset shows fast Fourier transform (FFT) pattern for selected area. (c) The EDS spectrum of $PdSe_2$ sample. (d-e) XPS results of the Pd 3d and Se 3d.

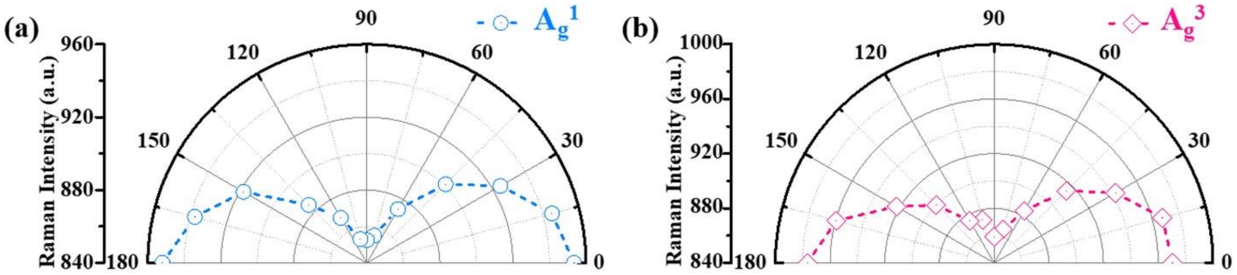

Figure S2 (a-b) Polar plots of Raman peak intensities of A_g^1 and A_g^3 mode as a function of polarization angle under parallel configurations.

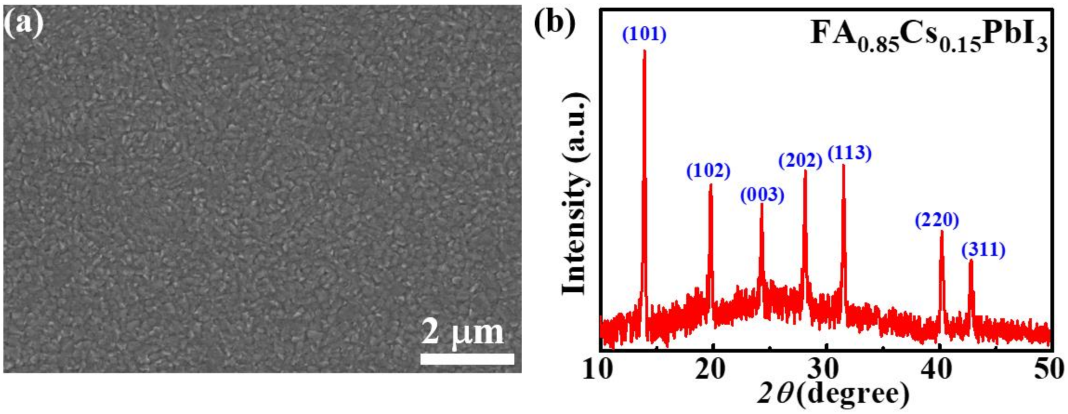

Figure S3 The FESEM image (a) and XRD pattern (b) of $FA_{0.85}Cs_{0.15}PbI_3$ perovskite films.

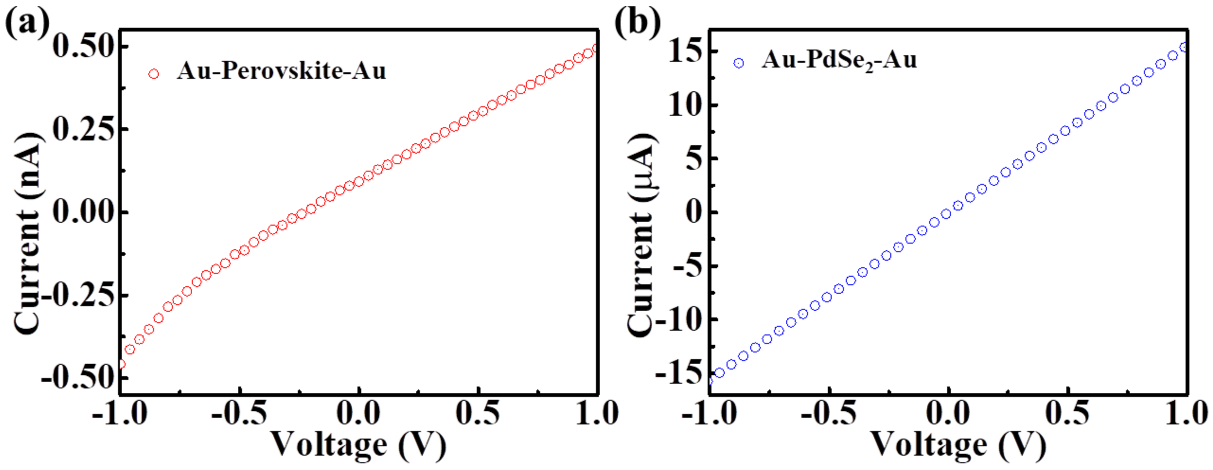

Figure S4 *I-V* curves for Au-perovskite-Au (a) and Au-$PdSe_2$-Au structure, indicating the good Ohmic contacts.

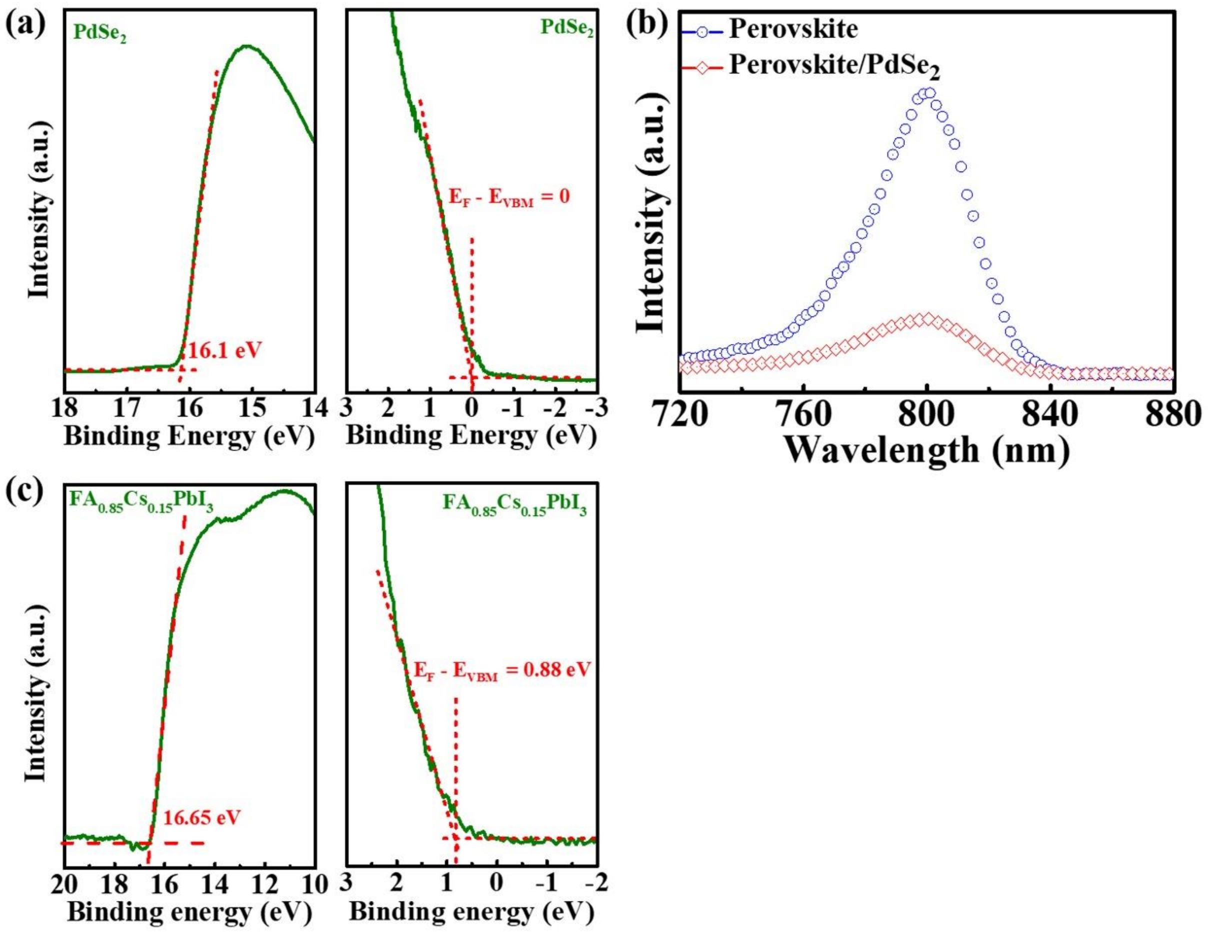

Figure S5 (a) UPS spectrum of multilayer $PdSe_2$ films. Energy difference between the Fermi level (E_F) and valence band maximum (E_{VBM}) extracted by UPS analysis, indicating that as-obtained $PdSe_2$ sample is semimetal. (b) PL spectrum of $FA_{0.85}Cs_{0.15}PbI$ perovskite and and $PdSe_2$/perovskite hybrid system. (c) UPS spectrum of $FA_{0.85}Cs_{0.15}PbI$ perovskite.

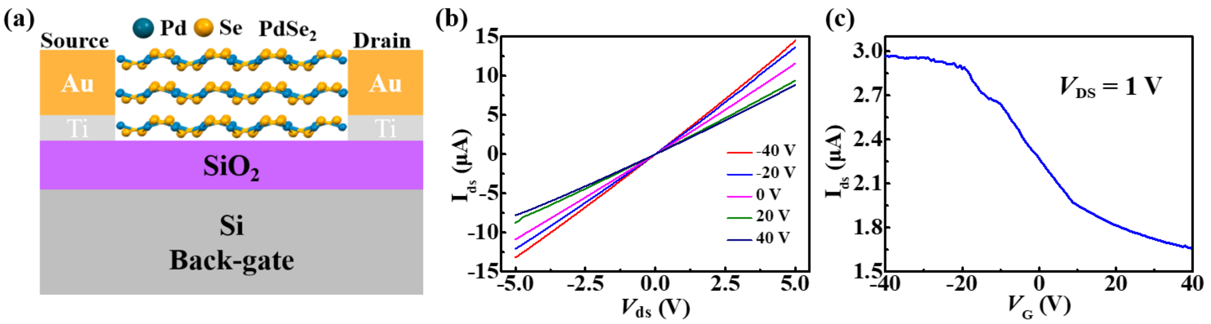

Figure S6 (a) Schematic illustration of multilayer $PdSe_2$ thin film field effect transistor. (b-c) The output and transfer characteristics of a typical $PdSe_2$ field-effect transistor.

Calculation of carrier mobility of the multilayer $PdSe_2$ thin film: To determine carrier mobility of as-fabricated $PdSe_2$ films, the back-gate field-effect transistors (FETs) based on multilayer $PdSe_2$ films have been fabricated on SiO_2/Si substrate as shown in Figure S5a. The typical output and transfer characteristics of as-fabricated FETs show a decreased drain current with positive gate voltage suggesting the typical p-type transport behavior of $PdSe_2$ films (Figure S6(b-c)). From the linear region in the transfer curve, the hole mobility of $PdSe_2$ films can be calculated using the equation: $\mu = \frac{1}{C_g}\frac{L}{W}\frac{1}{V_{ds}}\frac{dI_{ds}}{dV_g}$, where L= 100 μm is channel length, W = 100 μm is channel width, dI_{ds}/dV_g is the transconductance, and C_g is the capacitance of the dielectric ($C_g = \varepsilon_0\varepsilon_r/d$, ε_0 and ε_r denote the vacuum permittivity and relative permittivity of SiO_2, and d = 300 nm is the thickness of the SiO_2. Based on above equation, the hole mobility is calculated to be 4.75 $cm^2\ s^{-1}V^{-1}$.
